# Augmented glycaemic gap is a marker for an increased risk of post-infarct left ventricular systolic dysfunction

**DOI:** 10.1186/s12933-020-01075-8

**Published:** 2020-07-04

**Authors:** Yong Zhu, Kesen Liu, Shuai Meng, Ruofei Jia, Xuan Lei, Maolin Chen, Kaiyuan Zou, Huagang Zhu, Zening Jin

**Affiliations:** 1 Department of Cardiology, Beijing Anzhen Hospital, Capital Medical University, Beijing, 100029 China; 2 Department of Cardiology, Beijing Tiantan Hospital, Capital Medical University, Beijing, China

**Keywords:** Left ventricular systolic dysfunction, Change in LVEF, Stress-induced hyperglycaemia, Glycaemic gap, Admission blood glucose, ST-segment elevation myocardial infarction

## Abstract

**Background:**

Left ventricular systolic dysfunction (LVSD) occurs frequently after acute ST-segment elevation myocardial infarction (STEMI). The predisposing factors and underlying mechanism of post-infarct LVSD are not fully understood. The present study mainly investigated the correlation between glycaemic gap, a novel index of stress-induced hyperglycaemia (SIH), and post-infarct LVSD.

**Methods:**

A total of 274 first STEMI patients were enrolled in this cross-sectional study. Transthoracic echocardiography was performed within 48 h after admission and at 6 months after discharge to obtain left ventricular ejection fraction (LVEF). The change in LVEF was calculated as LVEF at 6 months after discharge minus baseline LVEF. Additionally, post-infarct LVSD was defined as LVEF ≤ 50%. Most importantly, glycaemic gap was calculated as admission blood glucose (ABG) minus the estimated average glucose over the previous 3 months.

**Results:**

In patients without diabetes mellitus (DM), multivariate linear regression analysis revealed that both glycaemic gap (Beta = − 1.214, 95% CI − 1.886 to − 0.541, p < 0.001) and ABG (Beta = − 1.124, 95% CI − 1.795 to − 0.453, p = 0.001) were associated with change in LVEF. In DM patients, only glycaemic gap was still associated with change in LVEF, although this association was not observed in univariate linear regression analysis. Regarding the association between SIH and post-infarct LVSD, multivariate logistic regression analysis revealed that both glycaemic gap (OR = 1.490, 95% CI 1.043 to 2.129, p = 0.028) and ABG (OR = 1.600, 95% CI 1.148 to 2.229, p = 0.005) were associated with an increased risk of having post-infarct LVSD in non-DM patients. However, after multivariate adjustment in DM patients, only glycaemic gap (OR = 1.399, 95% CI 1.021 to 1.919, p = 0.037) remained associated with an increased risk of having post-infarct LVSD. Furthermore, the predictive value of glycaemic gap for post-infarct LVSD was not inferior to ABG in non-DM patients (p = 0.499), and only glycaemic gap, instead of ABG, could significantly predict post-infarct LVSD in DM patients (AUC = 0.688, 95% CI 0.591 to 0.774, p = 0.002).

**Conclusions:**

Glycaemic gap was strongly associated with a change in LVEF and an increased risk of having post-infarct LVSD in patients following STEMI. In STEMI patients with DM, glycaemic gap could provide more valuable information than ABG in identifying patients at high risk of developing post-infarct LVSD.

## Background

Despite rapid development in acute treatment and second prevention in recent years, post-infarct left ventricular systolic dysfunction (LVSD) occurs frequently [1, 2]. Post-infarct LVSD, which is defined as reduced left ventricular ejection fraction (LVEF), is strongly correlated with adverse cardiovascular outcomes such as heart failure (HF), re-infarction, and increased cardiovascular mortality [3–6].

The pathophysiology of post-infarct LVSD is complex and poor understood [5]. Much evidence has revealed that processes such as myocardial injury associated with infarct size, left ventricular remodelling, myocardial stress, oxidative stress, and local and systemic inflammatory responses are all involved in the pathophysiology of post-infarct LVSD [5, 7–9]. Recent studies further indicated that stress-induced hyperglycaemia (SIH) and concomitant metabolic perturbations also play an extremely important role in the initiation and progression of post-infarct LVSD [10–12].

Critical illness such as acute myocardial infarction (AMI), sepsis, and major surgeries could lead to transient hyperglycaemia, termed SIH, through multiple potential mechanisms in patients with or without diabetes mellitus (DM) [13]. Admission blood glucose (ABG) has been mainly regarded as an index of SIH in previous studies [13]. The ABG level is impacted by both acute physiological stress and chronic baseline glycaemic levels, so it does not reflect the extent of SIH accurately in an acutely ill state [14, 15]. Furthermore, reports by Esposito et al. and Monnier et al. suggested that acute glucose fluctuations induced by SIH could be more harmful than sustained hyperglycaemia [16, 17]. To assess the effect of SIH more accurately, glycaemic gap and stress hyperglycaemia ratio (SHR), which eliminates the interference of chronic baseline glycaemic levels, were proposed as a novel index of SIH [18–20]. Considerable evidence have confirmed both glycaemic gap and SHR were strongly correlated with adverse cardiovascular outcome in AMI patients [15, 21, 22], but we knew little about the association between these indexes and post-infarct LVSD. Therefore, we mainly investigated the correlation between glycaemic gap and post-infarct LVSD in the present study.

## Methods

### Study population

In this cross-sectional study, we consecutively recruited ST-segment elevation myocardial infarction (STEMI) patients admitted to Beijing Anzhen Hospital, Capital Medical University from January 2018 to January 2019. Inclusion criteria were as follows: first STEMI diagnosed according to the European Society of Cardiology and American College of Cardiology Committee criteria [23, 24], emergent treatment of only the infarct-related artery by primary percutaneous coronary intervention (PCI) within 12 h, and other diseased vessels managed by elective PCI within 1 month if required. Exclusion criteria were defined as follows: age < 18 years or age > 80 years, previous history of myocardial infarction or coronary revascularization, thrombolysis prior to primary PCI, without stent implantation, serious valvular heart disease and primary cardiomyopathies, estimated glomerular filtration rate < 30 mL/min per 1.73 m^2^, active inflammatory or neoplastic process on admission, chronic requirement of steroid or immunosuppressive therapy. Additionally patients with hemopathy including hemolytic anemia, sickle cell anemia, thalassemia, and megaloblastic anemia were also excluded from our present study.

The study protocol was developed in compliance with the Declaration of Helsinki, and the study was approved by the Ethics Committee of Beijing Anzhen Hospital, Capital Medical University. Before the beginning of the study, we obtained written informed consent from each patient.

### Therapy and management

All enrolled patients received optimal treatment according to current guidelines for the management of acute STEMI patients [23, 24]. All the patients were prescribed a loading dose of aspirin 300 mg, clopidogrel 600 mg or ticagrelor 180 mg, and intravenously unfractionated heparin (60–70 IU/kg, maximum 5000 IU) immediately after diagnosis of STEMI. In the cardiac catheterization laboratory, all patients received a second dose of unfractionated heparin according to weight (70–100 IU/kg). The experienced interventional cardiologist blinded to the study protocol performed the primary PCI in compliance with current guidelines [23, 24]. After primary PCI, dual antiplatelet therapy in the form of aspirin plus ticagrelor or clopidogrel was recommended for 1 year if there were no contraindications. In addition, concomitant medications such as beta-blockers, statins, and angiotensin-converting enzyme inhibitors/angiotensin receptor blockers (ACEIs/ARBs) were all recommended unless contraindicated. Most importantly, lifestyle interventions, including smoking cessation, weight control, optimal blood pressure and blood glucose control, were also recommended.

### Data collection

When patients reached the emergency department, peripheral venous blood samples were collected immediately to measure ABG using a standardized biochemical assay. High-performance liquid chromatography analysers were used to examine glycated haemoglobin (HbA_1_c) levels. Then, we obtained the estimated average glucose (eAG) level over the previous 3 months through the following equation: eAG = 28.7 * HbA1c − 46.7 [14, 15, 18], and glycaemic gap was calculated as ABG minus eAG. SHR, another index of relative hyperglycaemia, was defined as ABG/eAG.

For biomarker analyses, cardiac troponin I (cTnI) and hypersensitive C-reactive protein (hs-CRP) were monitored dynamically to identify peak values. The other biological parameters, such as uric acid, homocysteine, triglyceride, low-density lipoprotein cholesterol (LDL-c), and high-density lipoprotein cholesterol (HDL-c), were all measured in a fasting state.

Demographic and clinical information, including age, sex, height, weight, smoking, DM, hypertension, and medication use at discharge, were all obtained through the standard questionnaire. In the questionnaire, DM was defined as having a prior history of DM (treated with diet or anti-diabetic medications) or having newly diagnosed DM with fasting plasma glucose ≥ 7.0 mmol/L, HbA_1_c ≥ 6.5% or 2-h plasma value ≥ 11.1 mmol/L during an oral glucose tolerance test [25]. Coronary angiogram data such as number of diseased vessels (stenosis ≥ 50% of diameter of coronary artery), thrombolysis in myocardial infarction (TIMI) flow grade pre- and post-PCI, and length of stents were all recorded by two independent cardiologists. Additionally, we defined slow flow/no-reflow as TIMI flow ≤ 2 post-PCI.

### Echocardiography

All patients included underwent examination of transthoracic echocardiography within 48 h after admission and 6 months after discharge to obtain LVEF with the modified Simpson rule. Transthoracic echocardiography was performed following recommendations from the American Society of Echocardiography [26]. Most importantly, transthoracic echocardiography was performed by the same two independent echocardiographers who have worked more than 5 years and were blinded to the study protocol with a GE Vivid 7 ultrasound machine (GE Healthcare, Piscataway, NJ, USA). Change in LVEF was defined as LVEF at 6 months post-STEMI minus baseline LVEF. Post-infarct LVSD was defined as LVEF ≤ 50%.

### Statistics

For continuous variables, a normal distribution was assessed using the Kolmogorov–Smirnov test. Then, continuous data were expressed as the mean ± standard deviation or median (interquartile ranges) and compared by Student’s t-test or the Mann–Whitney U test. Categorical variables were summarized as numbers (percentages) and analysed by the Chi-square test. To interrogate the association between change in LVEF and other variables, multivariate linear regression analysis was implemented (age, sex, total ischaemic time, number of diseased vessels, and variables with p < 0.1 in the univariate linear regression were included in the model). Additionally, the correlation between post-infarct LVSD and other variables was determined by multivariate logistic regression analysis (the model included age, sex, and variables with p < 0.1 in the univariate logistic regression). Furthermore, we assessed the predictive value of glycaemic gap and ABG through receiver operating characteristic (ROC) curve analysis. The predictive values of glycaemic gap and ABG were evaluated by the area under curve (AUC), and differences in the AUC were assessed by the DeLong test. Statistical analyses were performed using SPSS 20.0 (SPSS., Chicago, IL, USA) and MedCalc V.11.4 (MedCalc, Inc., Ostend, Belgium). p < 0.05 was regarded as statistically significant.

## Results

### Basic characteristics of the studied population

A total of 274 STEMI patients were enrolled for the final analyses in the present study after excluding 5 patients who died within 6 months after primary PCI and 3 patients who did not attend the 6-month visit. The patients enrolled were divided into 2 subgroups (non-DM group and DM group). Then, the non-DM (0.995 mmol/L) and DM (2.427 mmol/L) groups were further divided according to the median glycaemic gap level.

Table 1 shows that the mean age of the patients recruited was 57.78 years, and the prevalence of DM, current smoking, and hypertension was 39.1%, 65.7%, and 54.0%, respectively. Furthermore, as revealed in Additional file 1: Table S1, 48.5% of the patients had culprit vessel lesions in the left anterior descending artery (LAD), and 60.2% of the patients had multivessel disease. Additionally, as demonstrated in Additional file 1: Table S1, 94.5% of patients achieved TIMI flow grade 3 after intervention, which means that almost all patients recruited received timely and successful primary PCI.

**Table 1:** Baseline characteristics

Clinical information	Total (274)	Non-DM, n = 167		p-value	DM, n = 107		p-value
		Group 1 (84)	Group 2 (83)		Group 3 (54)	Group 4 (53)	
Age (years)	57.78 ± 11.42	55.18 ± 12.49	58.19 ± 9.95	0.087	58.54 ± 10.60	60.49 ± 12.08	0.376
Male	236 (86.1%)	77 (91.7%)	70 (84.3%)	0.160	44 (81.5%)	45 (84.9%)	0.797
BMI (kg/m^2^)	25.29 ± 2.62	25.34 ± 2.20	25.22 ± 2.84	0.754	25.51 ± 2.58	25.10 ± 2.94	0.441
Smoker	180 (65.7%)	59 (70.2%)	53 (63.9%)	0.413	33 (61.1%)	35 (66.0%)	0.689
DM	107 (39.1%)	–	–	–	54 (100.0%)	53 (100.0%)	1.000
Hypertension	148 (54.0%)	48 (57.1%)	40 (48.2%)	0.280	29 (53.7%)	31 (58.5%)	0.698
Killip classification				0.245			0.309
Class I	255 (93.1%)	75 (89.3%)	80 (96.4%)		52 (96.3%)	48 (90.6%)	
Class II	13 (4.7%)	6 (7.1%)	2 (2.4%)		2 (3.7%)	3 (5.7%)	
Class III	2 (0.7%)	1 (1.2%)	1 (1.2%)		–	–	
Class IV	4 (1.5%)	2 (2.4%)	0		0	2 (3.8%)	
Laboratory examination							
Uric acid (μmol/L)	359.70 ± 86.43	377.26 ± 91.46	356.86 ± 79.79	0.126	338.67 ± 95.03	357.74 ± 75.17	0.252
Homocysteine (μmol/L)	12.50 (9.38, 17.43)	14.25 (10.00, 23.35)	12.90 (9.30, 18.10)	0.133	11.05 (9.18, 15.25)	12.30 (9.15, 16.60)	0.413
eGFR (mmol/L)	99.56 (91.58, 107.78)	99.44 ± 15.01	98.19 ± 13.19	0.571	99.70 ± 14.06	94.18 ± 17.80	0.077
Tg (mmol/L)	1.45 (1.01, 2.05)	1.52 (1.06, 2.03)	1.29 (0.78, 1.70)	0.016	1.76 ± 0.93	2.48 ± 2.08	0.022
LDL-C (mmol/L)	3.14 ± 0.98	3.26 ± 1.05	3.19 ± 0.93	0.555	2.95 ± 0.83	3.07 ± 1.08	0.517
HDL-C (mmol/L)	1.05 (0.88, 1.20)	1.07 ± 0.25	1.13 ± 0.28	0.156	0.98 ± 0.21	1.06 ± 0.31	0.094
Peak cTnI (ng/mL)	30.80 (12.40, 68.52)	22.35 (10.20, 48.77)	40.97 (14.26, 75.00)	0.019	33.38 ± 30.59	47.47 ± 31.88	0.022
Peak hs-CRP (mg/L)	4.75 (2.27, 10.09)	4.72 (2.50, 7.58)	4.93 (1.37, 11.05)	0.804	4.53 (2.25, 7.98)	5.29 (2.86, 15.36)	0.323
ABG (mmol/L)	8.35 (7.02, 10.84)	6.59 ± 0.64	8.66 ± 1.47	< 0.001	10.03 ± 2.49	13.88 ± 2.95	<0.001
HBA_1_C (%)	6.00 (5.58, 6.90)	5.71 ± 0.35	5.62 ± 0.40	0.139	7.64 ± 1.56	7.48 ± 1.31	0.556
Pre-hospital medications							
Insulin	11 (4.0%)	–	–		4 (7.4%)	7 (13.2%)	0.359
DPP-4 inhibitors	22 (8.9%)	–	–		9 (16.7%)	13 (24.5%)	0.347
Other hypoglycemic agents	62 (22.7%)	–	–		34 (63.0%)	28 (52.8%)	0.331
Medications at discharge							
Aspirin	274 (100.0%)	84 (100.0%)	83 (100.0%)	1.000	54 (100.0%)	53 (100.0%)	1.000
Clopidogrel/ticagrelor	274 (100.0%)	84 (100.0%)	83 (100.0%)	1.000	54 (100.0%)	53 (100.0%)	1.000
Statin	270 (98.5%)	84 (100.0%)	80 (96.4%)	0.121	54 (100.0%)	52 (98.1%)	0.495
Beta-blockers	215 (78.5%)	68 (81.0%)	61 (73.5%)	0.273	45 (83.3%)	41 (77.4%)	0.474
ACEI/ARB	175 (63.9%)	52 (61.9%)	48 (57.8%)	0.637	38 (70.4%)	37 (69.8%)	1.000

Non-DM and DM patients were further divided into subgroups according to median value of glycemic gap (Group 1: glycemic gap ≤ 0.995 mmol/L; Group 2: glycemic gap > 0.995 mmol/L; Group 3: glycemic gap ≤ 2.427 mmol/L; Group 4: glycemic gap > 2.427 mmol/L)

*DM* diabetes mellitus, *BMI* body mass index, *eGFR* estimated glomerular filtration rate, *Tg* triglycerides, *LDL*-*C* low-density lipoprotein cholesterol, *HDL*-*C* high-density lipoprotein cholesterol, *cTnI* cardiac troponin I, *hs*-*CRP* hypersensitive C-reactive protein, *ABG* admission blood glucose, *FBG* fasting blood glucose, *HBA*_*1*_*C* glycated hemoglobin, *DPP*-*4 inhibitors* dipeptidyl peptidase-4 inhibitors, *ACEI/ARB* angiotensin-converting enzyme inhibitor/angiotensin receptor blocker

After subgroup analysis, we discovered that STEMI patients with high glycaemic gaps in the non-DM (40.97 (14.26, 75.00) vs. 22.35 (10.20, 48.77), p = 0.019) and DM (47.47 ± 31.88 vs. 33.38 ± 30.59, p = 0.022) groups both tended to have higher peak cTnI levels. High glycaemic gaps in non-DM (8.66 ± 1.47 vs. 6.59 ± 0.64, p < 0.001) and DM (13.88 ± 2.95 vs. 10.03 ± 2.49, p < 0.001) patients were also associated with higher ABG levels. Additionally, high glycaemic gap in DM patients was also associated with higher triglyceride (2.48 ± 2.08 vs. 1.76 ± 0.93, p = 0.022) levels, but not in non-DM patients.

### Glycaemic gap associated with change in LVEF

**Fig. 1:**
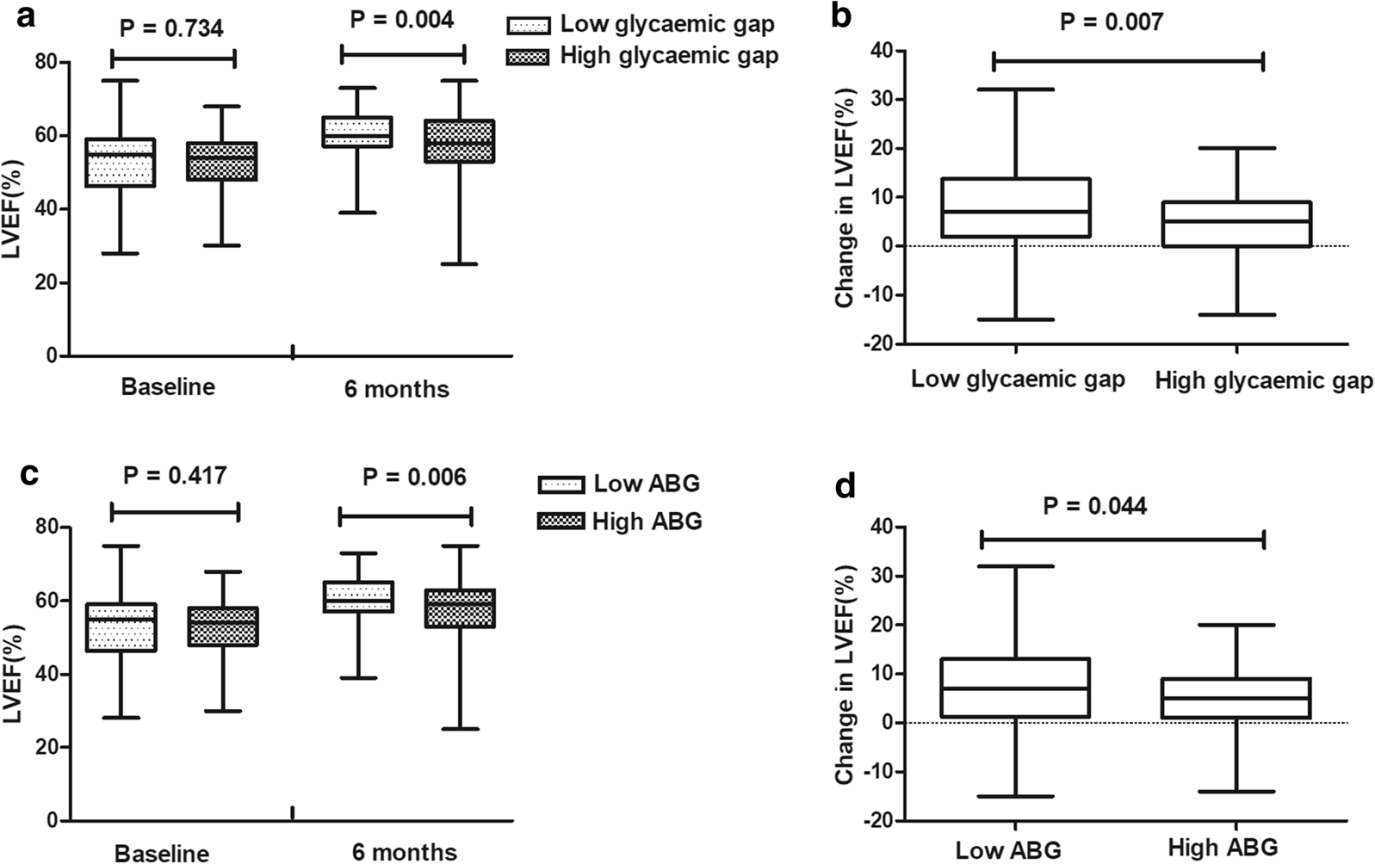
LVEF (**a**) and change in LVEF (**b**) in non-DM patients in the high glycaemic gap group and low glycaemic gap group at 6-month follow-up. LVEF (**c**) and change in LVEF (**d**) in non-DM patients in the high ABG group and low ABG group at 6-month follow-up. *LVEF* left ventricular ejection fraction, *DM* diabetes mellitus, *ABG* admission blood glucose. Change in LVEF was defined as LVEF at 6 months post-STEMI minus baseline LVEF

In non-DM patients, compared with the low glycaemic gap group, the high glycaemic gap group was associated with lower LVEF at 6 months post-STEMI (56.96 ± 9.12 vs. 60.55 ± 6.38, p = 0.004, Fig. 1a), and the change in LVEF (4.29 ± 6.82 vs. 7.50 ± 8.33, p = 0.007, Fig. 1b) was also significantly smaller in the high glycaemic gap group patients, despite the baseline LVEF (52.67 ± 7.71 vs. 53.11 ± 8.65, p = 0.734) being similar. Additionally, as presented in Fig. 1c, d, both the LVEF at the 6-month follow-up (57.06 ± 9.11 vs. 60.45 ± 6.44, p = 0.006) and the change in LVEF (4.69 ± 6.87 vs. 7.11 ± 8.42, p = 0.044) were significantly lower in the high ABG group than in the low ABG group in non-DM patients. Most importantly, as shown in Table 2, glycaemic gap (Beta = − 1.214, 95% CI − 1.886 to − 0.541, p < 0.001) and ABG (Beta = − 1.124, 95% CI − 1.795 to − 0.453, p = 0.001) remained associated with the change in LVEF in non-DM patients after adjusting for age, sex, BMI, peak hs-CRP, baseline LVEF, total ischaemic time, and number of diseased vessels.

**Table 2:** Multivariate linear regression for the correlation between change in LVEF and other variables in non-DM

Non-DM patients	Univariate linear regression			Multivariate linear regression (Mode A, R^2^ = 0.379)			Multivariate linear regression (Mode B, R^2^ = 0.373)		
Variables	Beta	95% CI	p-value	Beta	95% CI	p-value	Beta	95% CI	p-value
Age	− 0.029	− 0.134 to 0.076	0.582	0.077	− 0.015 to 0.169	0.099	0.098	0.004 to 0.193	0.042
Sex	− 3.811	− 7.428 to − 0.193	0.039	− 4.107	− 7.250 to − 0.964	0.011	− 4.337	− 7.490 to − 1.183	0.007
BMI	0.463	− 0.003 to 0.928	0.051	0.286	− 0.109 to 0.680	0.155	0.318	− 0.078 to 0.714	0.115
Peak hs-CRP	− 0.152	− 0.304 to 0.001	0.051	− 0.185	− 0.312 to − 0.058	0.005	− 0.190	− 0.318 to − 0.063	0.004
Baseline LVEF	− 0.469	− 0.596 to − 0.342	< 0.001	− 0.492	− 0.614 to − 0.370	< 0.001	− 0.498	− 0.621 to − 0.375	< 0.001
Total ischemic time	− 0.004	− 0.012 to 0.004	0.318	− 0.005	− 0.011 to 0.001	0.114	− 0.005	− 0.011 to 0.002	0.148
No. of diseased vessels	− 0.516	− 1.958 to 0.927	0.481	− 0.171	− 1.364 to 1.022	0.778	− 0.174	− 1.374 to 1.026	0.775
ABG	− 0.866	− 1.634 to − 0.099	0.027				− 1.124	− 1.795 to − 0.453	0.001
Glycemic gap	− 1.116	− 1.901 to − 0.331	0.006	− 1.214	− 1.886 to − 0.541	< 0.001			

Change in LVEF was defined as LVEF at 6-month follow-up minus baseline LVEF

*DM* diabetes mellitus, *BMI* body mass index, *hs*-*CRP* hypersensitive C-reactive protein, *LVEF* left ventricular ejection fraction, *Total ischemic time* the period from symptom onset to reopening of infarction-associated artery, *ABG* admission blood glucose

**Fig. 2:**
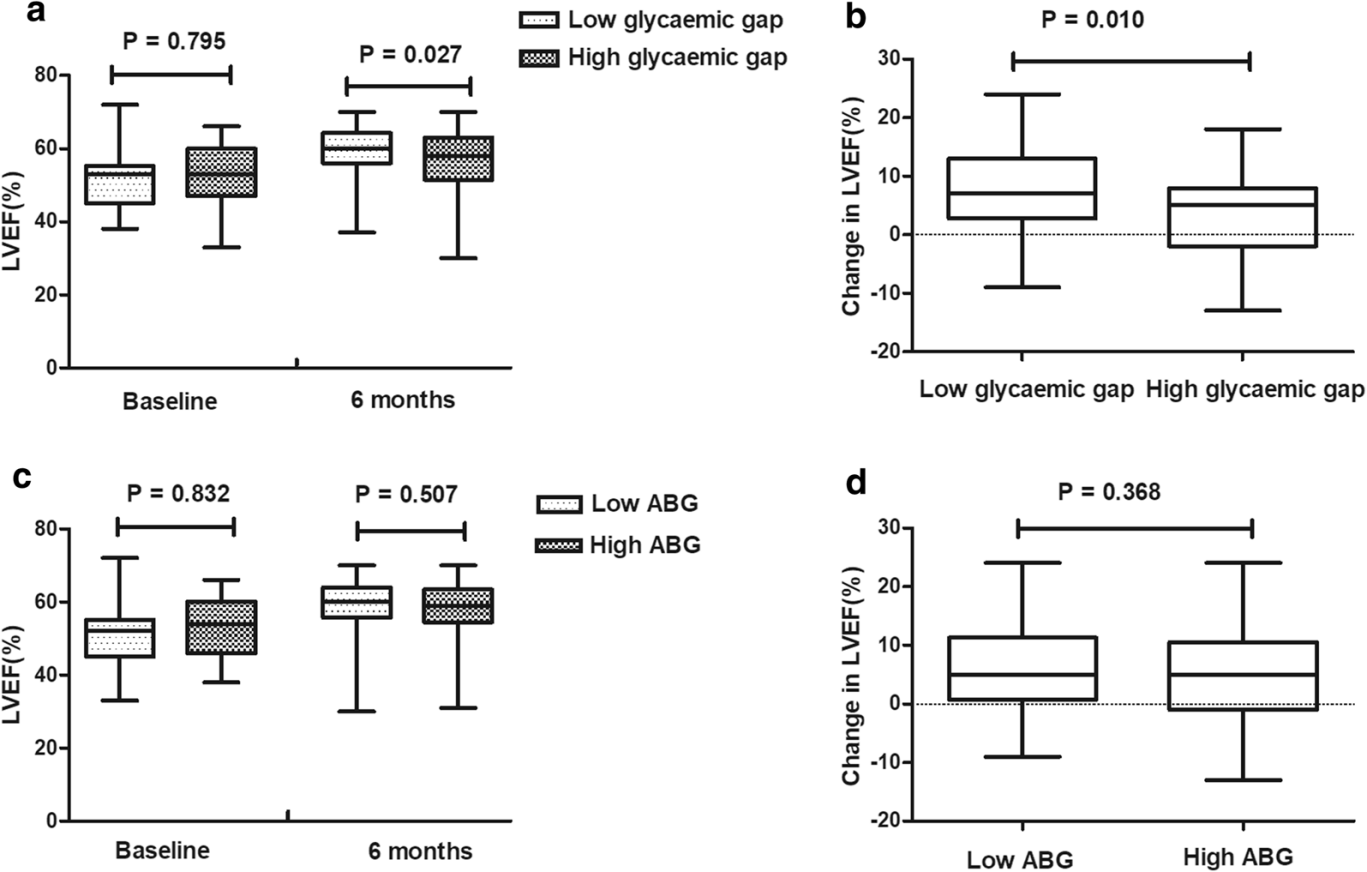
LVEF (**a**) and change in LVEF (**b**) in DM patients in the high glycaemic gap group and low glycaemic gap group at 6-month follow-up. LVEF (**c**) and change in LVEF (**d**) in DM patients in the high ABG group and low ABG group at 6-month follow-up. *LVEF* left ventricular ejection fraction, *DM* diabetes mellitus, *ABG* admission blood glucose. Change in LVEF was defined as LVEF at 6 months post-STEMI minus baseline LVEF

As demonstrated in Fig. 2a, b, a high glycaemic gap was still associated with lower LVEF (56.13 ± 9.38 vs. 59.61 ± 6.28, p = 0.027) and change in LVEF (3.43 ± 7.80 vs. 7.31 ± 7.51, p = 0.010) at the 6-month follow-up in DM patients. However, as presented in Fig. 2c, d, we observed no difference in LVEF and change in LVEF between the high ABG group and the low ABG group in DM patients at the 6-month follow-up. In DM patients, univariate linear regression revealed that both glycaemic gap (Beta = − 0.366, 95% CI − 0.942 to 0.210, p = 0.210) and ABG (Beta = − 0.281, 95% CI − 0.734 to 0.172, p = 0.221) were not correlated with change in LVEF.

### Glycaemic gap was strongly correlated with post-infarct LVSD

**Fig. 3:**
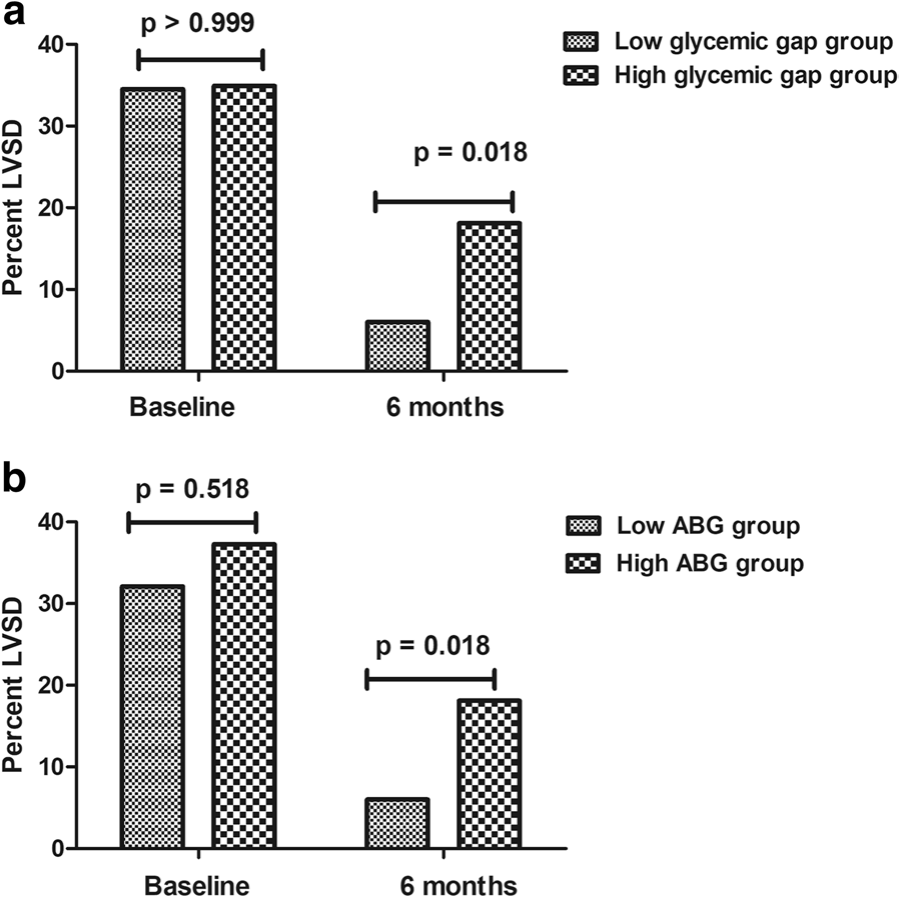
The proportion of post-infarct LVSD in non-DM patients in the high glycaemic gap group and low glycaemic gap group at baseline and 6-month follow-up (**a**). The proportion of post-infarct LVSD in non-DM patients in the high ABG group and low ABG group at baseline and 6-month follow-up (**b**). *LVSD* left ventricular systolic dysfunction, *DM* diabetes mellitus, *ABG* admission blood glucose. Post-infarct LVSD was defined as LVEF ≤ 50% after infarction

In non-DM patients, the proportion of post-infarct LVSD in high glycaemic gap patients was significantly higher than that in low glycaemic gap patients at the 6-month follow-up (18.1% vs. 6.0%, p = 0.018, Fig. 3a), although the proportion of post-infarct LVSD was similar between subgroups at baseline (34.9% vs. 34.5%, p > 0.99, Fig. 3a). Additionally, as revealed in Fig. 3b, post-infarct LVSD at the 6-month follow-up also occurred more often in the high ABG group patients (18.1% vs. 6.0%, p = 0.018). Furthermore, as revealed in Table 3, multivariate logistic regression revealed that an augmented glycaemic gap (OR = 1.490, 95% CI 1.043 to 2.129, p = 0.028) and ABG (OR = 1.600, 95% CI 1.148 to 2.229, p = 0.005) were associated with an increased risk of having post-infarct LVSD after adjusting for age, sex, peak cTnI, HDL-c, and peak hs-CRP in non-DM patients.

**Table 3:** Multivariate logistic regression for the correlation between post-infarct LVSD and other variables in non-DM and DM patients

Non-DM patients	Univariate logistic regression			Multivariate logistic regression (Mode A)			Multivariate logistic regression (Mode B)		
Variables	OR	95% CI	p-value	OR	95%	p-value	OR	95%	p-value
Age	0.986	0.947 to 1.027	0.494	0.950	0.897 to 1.005	0.073	0.939	0.884 to 0.996	0.038
Sex	0.341	0.109 to 1.071	0.065	0.216	0.046 to 1.017	0.053	0.195	0.041 to 0.934	0.041
Peak cTnI	1.025	1.008 to 1.042	0.003	1.015	0.997 to 1.033	0.106	1.015	0.997 to 1.034	0.111
HDL-C	5.707	1.167 to 27.919	0.032	3.972	0.600 to 26.284	0.153	4.626	0.679 to 31.509	0.118
Peak hs-CRP	1.074	1.020 to 1.131	0.006	1.070	1.010 to 1.135	0.022	1.075	1.013 to 1.141	0.017
ABG	1.499	1.146 to 1.962	0.003				1.600	1.148 to 2.229	0.005
Glycemic gap	1.494	1.120 to1.993	0.006	1.490	1.043 to 2.129	0.028			

DM patients	Univariate logistic regression			Multivariate logistic regression					
Variables	OR	95% CI	p-value	OR	95% CI	p-value			
Age	1.028	0.977 to 1.081	0.287	1.013	0.945 to 1.085	0.722			
Sex	3.038	0.894 to 10.326	0.075	0.317	0.057 to 1.780	0.192			
Peak cTnI	1.048	1.023 to 1.073	< 0.001	1.051	1.023 to 1.080	< 0.001			
HDL-C	6.183	0.975 to 39.221	0.053	0.588	0.073 to 4.736	0.617			
Peak hs-CRP	1.025	0.964 to 1.090	0.434						
ABG	1.106	0.947 to 1.292	0.203						
Glycemic gap	1.267	1.027 to 1.565	0.027	1.399	1.021 to 1.919	0.037			

LVSD was defined as LVEF ≤ 50%

*LVSD* left ventricular systolic dysfunction, *DM* diabetes mellitus, *cTnI* cardiac troponin I, *HDL*-*C* high-density lipoprotein cholesterol, *hs*-*CRP* hypersensitive C-reactive protein, *ABG* admission blood glucose

**Fig. 4:**
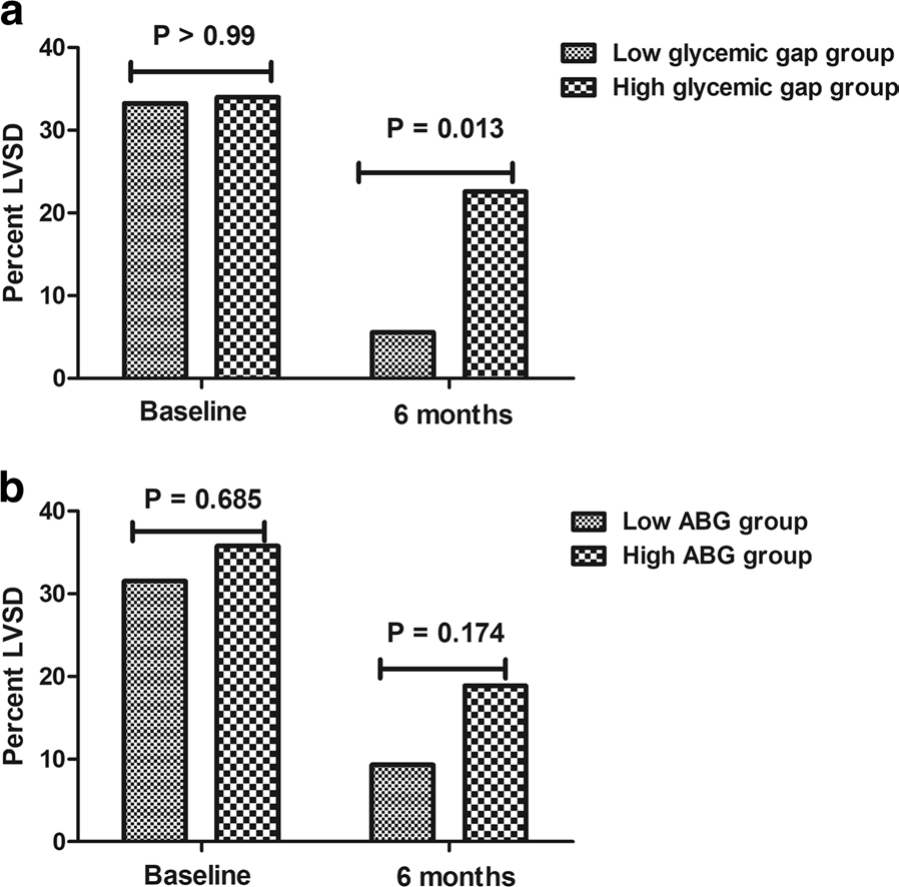
The proportion of post-infarct LVSD in DM patients in the high glycaemic gap group and low glycaemic gap group at baseline and 6-month follow-up (**a**). The proportion of post-infarct LVSD in DM patients in the high ABG group and low ABG group at baseline and 6-month follow-up (**b**). *LVSD* left ventricular systolic dysfunction, *DM* diabetes mellitus, *ABG* admission blood glucose. Post-infarct LVSD was defined as LVEF ≤ 50% after infarction

As shown in Fig. 4a, although the proportion of post-infarct LVSD at baseline was similar between subgroups in DM patients, high glycaemic gap patients were still associated with a higher proportion of post-infarct LVSD at the 6-month follow-up compared with low glycaemic gap patients (22.6% vs. 5.6%, p = 0.013). In line with this finding, as indicated in Table 3, univariate logistic regression revealed that an elevated glycaemic gap was strongly associated with an increased risk of having post-infarct LVSD at 6 months post-STEMI (OR = 1.267, 95% CI 1.027 to 1.565, p = 0.027). After adjusting for age, sex, peak cTnI, and HDL-c through multivariate logistic regression, the correlation persisted in DM patients (OR = 1.399, 95% CI 1.021 to 1.919, p = 0.037). However, as indicated in Fig. 4b, there were no significant differences between the high ABG and low ABG groups in the prevalence of post-infarct LVSD at baseline and 6 months post-STEMI. Additionally, univariate logistic regression also revealed that ABG also was not correlated with post-infarct LVSD at the 6-month follow-up (OR = 1.106, 95% CI 0.947 to 1.292, p = 0.203).

### Predictive value of glycaemic gap compared to ABG for post-infarct LVSD at the 6-month follow-up

After ROC analysis (Table 4), we demonstrated that both glycaemic gap (AUC = 0.697, 95% CI 0.622 to 0.766, p = 0.001) and ABG (AUC = 0.713, 95% CI 0.638 to 0.781, p = 0.002) could provide a moderate predictive value for post-infarct LVSD at the 6-month follow-up, and the predictive value of glycaemic gap was not inferior to ABG (p = 0.499) in non-DM patients. However, as shown in Table 4, only glycaemic gap had a moderate predictive value for post-infarct LVSD at 6 months after STEMI (AUC = 0.688, 95% CI 0.591 to 0.774, p = 0.002) in DM patients.

**Table 4:** Predictive value of glycemic gap versus ABG for post-infarct LVSD at 6-month follow-up

	AUC	95% CI	p for AUC	P for δAUC	Cut-off value	Sensitivity	Specificity	PPV	NPV
Non-DM patients									
ABG	0.713	0.638 to 0.781	0.002	–	8.27	70.00%	78.23%	30.4%	95.0%
Glycemic gap	0.697	0.622 to 0.766	0.001	0.499	1.858	55.00%	78.91%	26.2%	92.8%
DM patients									
ABG	0.621	0.522 to 0.713	0.08						
Glycemic gap	0.688	0.591 to 0.774	0.002		2.197	86.67%	54.35%	23.6%	96.2%

*ABG* admission blood glucose, *LVSD* left ventricular systolic dysfunction, *DM* diabetes mellitus, *AUC* area under curve by receiver-operating characteristic curve analysis, *δ AUC* difference of AUC, *PPV* positive predictive values, *NPV* negative predictive values

## Discussion

Our present study demonstrated 3 significant findings. First, glycaemic gap, as a novel index of SIH, was associated with changes in LVEF and the increased risk of having post-infarct LVSD in STEMI patients with or without DM. Second, the correlation between change in LVEF, post-infarct LVSD, and ABG only persisted in non-DM patients. Third, in regard to identifying patients at high risk of having post-infarct LVSD, glycaemic gap could provide a superior discriminatory ability compared with ABG in DM patients.

Considerable evidences suggested glycaemic excursion is not only associated with risk of cardiovascular disease but also associated with major adverse cardiovascular and cerebrovascular events [27, 28]. Glycaemic gap, as a novel marker of glycaemic excursion that quantifies the magnitude of a relative glycaemic rise from chronic glycaemia in an acutely ill state, was proposed to better assess the effect of SIH [20]. Previous studies have reported glycaemic gap is associated with severity of disease and unfavorable prognosis in patients with critical illness [29]. Our present study further confirmed that peak cTnI was significantly higher in patients with a high glycaemic gap level regardless of diabetic status, which suggests SIH measured by glycaemic gap is associated with infarct size assessed by peak cTnI. According to previous studies, the potential mechanisms of the relationship between SIH and myocardial injury included secretion of excessive cortisol and catecholamine, inflammation and oxidative stress, endothelial dysfunction, relative insulin deficiency, and prothrombotic state [13, 16, 17, 30].

Recent clinical trials have confirmed ABG levels are associated with poor prognosis in STEMI patients without DM, but the association is relative weak in DM patients [31, 32]. However, the association between ABG and myocardial function after STEMI in patients with DM or without DM is not well investigated at present, and direct evidence is limited. Capes et al. reported that ABG was correlated with an increased risk of congestive heart failure or cardiogenic shock in non-DM patients, but the correlation was not observed in DM patients [31]. Teraguchi et al. further demonstrated that myocardial salvage index (MSI) assessed cardiac magnetic resonance (CMR) in patients with SIH (ABG ≥ 10 mmol/L) was lower than that in patients without SIH among non-DM patients; however, no significant difference was observed in MSI between patients with or without SIH in DM patients [33]. In line with these studies, our present study revealed that ABG was associated with changes in LVEF and the risk of having post-infarct LVSD in non-DM patients, but the association was not observed in DM patients. There may be several explanations for the discrepant findings. First, not taking baseline glucose levels into consideration in DM patients may be the main reason. There is a wide range in the level of chronic glycaemic control among DM patients, from satisfactory to poor; therefore, it is necessary to consider baseline glucose levels when investigating the correlation between SIH and the risk of post-infarct LVSD. Second, many DM patients achieve good glycaemic control with optimal glucose-lowering treatment, while others do not.

Instead of ABG, both glycaemic gap and SHR were associated with major adverse cardiac events (MACEs) and could provide moderate predictive value for the occurrence of MACEs in AMI patients with DM [15, 22, 34]. Liao et al. further demonstrated glycaemic gap rather than ABG was linked to all-cause mortality in acute heart failure (AHF) patients with DM [35]. Moreover, they also discovered glycaemic gap could provide higher predictive value than ABG for all-cause mortality, cardiovascular mortality, and acute respiratory failure [35]. Extending prior studies, our present study demonstrated for the first time that glycaemic gap, not ABG, was associated with the change in LVEF and risk of having post-infarct LVSD in DM patients. Comparing with ABG, glycaemic gap could provide a superior predictive ability for post-infarct LVSD in DM patients. Therefore, glycaemic gap could be used to assess prognosis and identify patients at high risk for developing post-infarct LVSD in the management of AMI patients, particularly in patients with DM. However, we should note that the accuracy of glycaemic gap is still moderate, and its performance should be further explored by prospective longitudinal studies before putting it into practice.

Additionally, Lee et al. reported that relative hyperglycaemia during glucose-lowering treatment is more strongly associated with adverse outcomes than absolute glycaemia in patients following AMI [36]. The results suggested that using relative, not absolute, glycaemic thresholds for intervention and therapeutic glycaemic targets could improve adverse outcomes following AMI [36]. Therefore, it is necessary for future randomized controlled trials to investigate whether a glucose-lowering treatment target on glycaemic gap or SHR could reduce the risk of developing post-infarct LVSD in STEMI patients.

We acknowledge that the present study has several limitations. First, this was a single-centre study with a limited sample size. Therefore, experimental and multi-center clinical studies with large sample size are needed to further verify our findings in the future. Second, this was an observational study, which failed to assess the causal link between the relative hyperglycaemia glycaemic gap and post-infarct LVSD. Third, we evaluated changes in LVEF and post-infarct LVSD only through transthoracic echocardiography, which may limit the value of our research. To improve accuracy and provide more valuable information, future studies could employ CMR or single-photon emission computed tomography to assess associated parameters including LVEF, infarct size, microvascular obstruction, and MSI. Finally, we only recruited STEMI patients who underwent primary PCI successfully in the present study. Thus, our findings remain to be verified in general patients with acute coronary syndrome.

## Conclusions

Our present study demonstrated for the first time that glycaemic gap, as a novel index of SIH, was associated with the change in LVEF and the risk of having post-infarct LVSD at the 6-month follow-up in STEMI patients after primary PCI. Compared to ABG, glycaemic gap could provide a superior predictive value for post-infarct LVSD in DM patients and an equivalent predictive value in non-DM patients. Prospective studies are needed to further investigate whether glycaemic control using glycaemic gap could reduce the risk of having post-infarct LVSD, especially in DM patients.

## Supplementary information

**Additional file 1: Table S1.** Procedural characteristics of patients enrolled.

## Data Availability

The datasets used and analysed during the current study are available from the corresponding author on reasonable request.

## Abbreviations

LVSD: Left ventricular systolic dysfunction
LVEF: Left ventricular ejection fraction
HF: Heart failure
SIH: Stress-induced hyperglycaemia
AMI: Acute myocardial infarction
DM: Diabetes mellitus
ABG: Admission blood glucose
SHR: Stress hyperglycaemia ratio
STEMI: ST-segment elevation myocardial infarction
PCI: Percutaneous coronary intervention
ACEIs/ARBs: Angiotensin-converting enzyme inhibitors/angiotensin receptor blockers
HbA1c: Glycated haemoglobin
eAG: Estimated average glucose
cTnI: Cardiac troponin I
hs-CRP: Hypersensitive C-reactive protein
LDL-c: Low-density lipoprotein cholesterol
HDL-c: High-density lipoprotein cholesterol
TIMI: Thrombolysis in myocardial infarction
ROC: Receiver operating characteristic
AUC: Area under curve
LAD: Left anterior descending artery
MSI: Myocardial salvage index
CMR: Cardiac magnetic resonance
MACEs: Major adverse cardiac events
AHF: Acute heart failure
