# Supplementary material for: Augmented glycaemic gap is a marker for an increased risk of post-infarct left ventricular systolic dysfunction
Source: Cardiovasc Diabetol. 2020 Jul 4;19:101. doi: 10.1186/s12933-020-01075-8 (PMC7335441; doi:10.1186/s12933-020-01075-8)
Supplement: Supplementary file 1 — Additional file 1: Table S1. Procedural characteristics of patients enrolled. [file 12933_2020_1075_MOESM1_ESM.docx]

Table S1: Procedural characteristics of patients enrolled.

|  | Total | Non-DM, n = 167 | | P-value | DM, n = 107 | | P-value |
| --- | --- | --- | --- | --- | --- | --- | --- |
|  |  | Group 1 (84) | Group 2 (83) |  | Group 3 (54) | Group 4 (53) |  |
| Infarcted related artery |  |  |  | 0.664 |  |  | 0.596 |
| LAD | 133(48.5%) | 42(50.0%) | 36(43.4%) |  | 30(55.6%) | 25(47.2%) |  |
| LCX | 28(10.2%) | 10(11.9%) | 10(12.0%) |  | 3(5.6%) | 5(9.4%) |  |
| RCA | 113(41.2%) | 32(38.1%) | 37(44.6%) |  | 21(38.9%) | 23(43.4%) |  |
| Number of diseased vessels |  |  |  | 0.916 |  |  | 0.406 |
| 1-vessel disease | 109(39.8%) | 36(42.9%) | 35(42.2%) |  | 17(31.5%) | 21(39.6%) |  |
| 2-vessel disease | 80(29.2%) | 26(31.0%) | 24(28.9%) |  | 14(25.9%) | 16(30.2%) |  |
| 3-vessel disease | 85(31.0%) | 22(26.2%) | 24(28.9%) |  | 23(42.6%) | 16(30.2%) |  |
| TIMI flow (pre-PCI) |  |  |  | 0.278 |  |  | 0.102 |
| Grade 0 | 210(76.6%) | 65(77.4%) | 63(75.9%) |  | 45(83.3%) | 37(69.8%) |  |
| Grade 1 | 44(16.1%) | 15(17.9%) | 11(13.3%) |  | 8(14.8%) | 10(18.9%) |  |
| Grade 2 | 20(7.3%) | 4(4.8%) | 9(10.8%) |  | 1(1.9%) | 6(11.3%) |  |
| Grade 3 | 0 | - | - |  | - | - |  |
| Total ischemic time (min) | 411.50(305.00,534.25) | 467.62±151.15 | 423.48±157.84 | 0.067 | 403.59±158.89 | 412.62±182.77 | 0.785 |
| Stent implantation | 274(100.0%) | 84(100.0%) | 83(100.0%) | 1.000 | 54(100.0%) | 53(100.0%) | 1.000 |
| Number of stents | 1.31±0.59 | 1.38±0.77 | 1.30±0.49 | 0.428 | 1.26±0.52 | 1.25±0.48 | 0.885 |
| Length of Stents (mm) | 30.00(22.00,38.00) | 30.00(20.00,42.50) | 30.00(20.00,48.00) | 0.763 | 32.50(24.00,36.00) | 30.00(20.50,36.00) | 0.325 |
| Thrombus aspiration | 125(45.6%) | 34(40.5%) | 42(50.6%) | 0.215 | 26(48.1%) | 23(43.4%) | 0.699 |
| GP IIb/IIIa inhibitor | 148(54.0%) | 46(54.8%) | 46(55.4%) | 1.000 | 29(53.7%) | 27(50.9%) | 0.847 |
| Slow flow/no-reflow | 15 (5.5%) | 5 (6.0%) | 3 (3.6%) | 0.720 | 3 (5.6%) | 4 (7.5%) | 0.716 |

DM, diabetes mellitus; LAD, left anterior descending artery; LCX, left circumflex artery; RCA , right coronary artery; TIMI, thrombolysis in myocardial infarction; PCI, percutaneous coronary intervention; Total ischemic time, the period from symptom onset to reopening of infarction-associated artery; GP IIb/IIIa, glycoprotein IIb/IIIa.

Non-DM and DM patients were further divided into subgroups according to median value of glycemic gap (Group 1: glycemic gap ≦ 0.995mmol/L; Group 2: glycemic gap > 0.995mmol/L; Group 3: glycemic gap ≦ 2.427mmol/L; Group 4: glycemic gap > 2.427mmol/L).
